# # Language processing characteristics in normal pressure hydrocephalus: insights from eye-tracking analysis of incorrect responses

**DOI:** 10.3389/fnagi.2025.1527962

**Published:** 2025-04-23

**Authors:** Ji-Yeong Kim, Jiho Lee, Nayeon Kim, Ki-Su Park, Janghyeok Yoon, Kyunghun Kang, Ji-Wan Ha

**Affiliations:** ^1^Department of Speech-Language Pathology, Daegu University, Gyeongsan, Republic of Korea; ^2^Neopons Inc., Daegu, Republic of Korea; ^3^Department of Neurosurgery, School of Medicine, Kyungpook National University, Daegu, Republic of Korea; ^4^Department of Industrial Engineering, Konkuk University, Seoul, Republic of Korea; ^5^Department of Neurology, School of Medicine, Kyungpook National University, Daegu, Republic of Korea

**Keywords:** NPH, eye-tracking, language processing, object naming, incorrect response, lexical error

## Abstract

**Introduction:**

In patients with Normal Pressure Hydrocephalus (NPH), the aging process results in decreased efficiency of cerebrospinal fluid circulation, leading to ventricular enlargement. This enlargement compresses several brain structures, impairing functions such as visual perception, semantic memory processing, and phonological encoding contributing to language processing difficulties. This study examines real-time language processing in NPH patients and healthy elderly (HE) controls by comparing their eye movements during correct and incorrect responses in a lexical retrieval task (LRT).

**Methods:**

A total of 26 subjects participated, comprising 14 patients diagnosed with NPH (aged 65 or older) and 12 individuals in the HE group. A lexical retrieval task was administered as their eye movements were recorded. A Mann–Whitney U test was performed to compare LRT performance and eye-tracking metric results across different groups, respectively. Also, correlation analysis was performed to examine the relationship between LRT scores and eye-tracking scores. A two-way mixed ANOVA was conducted to assess the significance of eye-tracking metrics depending on response type (correct/incorrect). Additionally, a qualitative and quantitative comparison of heatmaps and scanpaths was conducted to visualize eye-tracking data for correct and incorrect items.

**Results:**

The NPH group exhibited significantly lower performance in lexical retrieval compared to the HE group, accompanied by more counts and longer durations in both fixation and saccade metrics. A negative correlation was noted between LRT scores and eye-tracking metric values, with correlation coefficients predominantly at 0.50 or higher. Analysis of eye movements during correct and incorrect responses uncovered significant group and within-group effects across all metrics, with more intergroup differences during incorrect responses. Qualitative differences in eye movements were more noticeable in images associated with incorrect items.

**Discussion:**

This study highlights previously under-explored language deficits in NPH patients using real-time visual processing analysis, underscoring the importance of targeted language interventions for these populations.

## 1 Introduction

Most dementias involve progressive and irreversible deterioration of brain cells and their interconnections, but not all. Some dementias are reversible, suggesting the potential for recovery. Reversible dementia, when identified early, can show improvement or alleviation of symptoms, with normal pressure hydrocephalus (NPH) being one such condition (Mortazavi et al., 2022). The initial symptom of NPH is typically gait disturbance, followed by cognitive decline and urinary incontinence (Sinforiani et al., 2018). In NPH patients, impaired cerebrospinal fluid circulation, often associated with aging, causes ventricular enlargement. This enlargement compresses multiple brain structures, including the occipital, temporal, and frontal lobes, as well as the hippocampus. Such compression can impair functions such as visual perception, lexical access, phonological encoding, and semantic memory processing, contributing to language processing difficulties. Chung (2018) examined the records of 529 NPH patients who underwent shunt surgery and found that 23.1% exhibited language impairments, with expressive language deficits, especially in object naming, affecting approximately 75% of these individuals.

Object naming relies on visual recognition, semantic processing, lexical access, and phonological processing (Goodglass and Wingfield, 1997). Individuals with neurocognitive disorders often exhibit impairments in vocabulary, semantics, and visual perception, contributing to difficulties with object naming (Paek et al., 2011; Kim et al., 2024; Reilly et al., 2022). Despite the expectation that these functions may be weakened due to brain structure compression in NPH patients, research on this relationship remains remarkably limited. A recent study by Kim et al. (2024) compared the performance of NPH, Parkinson’s disease (PD), and normal elderly groups in a naming task, revealing that the NPH and PD groups performed significantly worse than the normal elderly group. Furthermore, the NPH group exhibited slower reaction times and produced more semantic and non-word errors. Conversely, Saito et al. (2011) found no significant differences in object naming performance between individuals with idiopathic NPH, Alzheimer’s disease (AD), and normal elderly individuals, highlighting the necessity for further research to resolve these conflicting results.

In patients with NPH, language processing deficits are thought to stem from broader cognitive impairments rather than a direct dysfunction of the language system itself (Whitehouse et al., 1993). Thus, depending on whether a task focuses on “static outcomes” (e.g., naming performance) or “dynamic processes” (e.g., naming processing), NPH patients may perform differently, with difficulties being more pronounced in the latter than in the former. Discrepancies in naming performance reported in previous studies (Kim et al., 2024; Saito et al., 2011) may reflect differences in the employed tasks. Consequently, this study investigates both static and dynamic aspects of naming tasks in NPH patients. To achieve this, the study employs a “lexical retrieval task,” suggested by Dell et al. (1997) and utilized by Kim et al. (2024), instead of the traditional object naming task. Traditional naming tasks assess the quantity of vocabulary or the final success rate, while lexical retrieval tasks examine the underlying retrieval processes. This task incorporates numerous low-frequency, multi-syllabic words to ensure a sensitive evaluation. Participants must respond immediately with the word that comes to mind upon viewing a picture, with no cues provided, and a point is awarded only if the initial response is correct. Even if a participant self-corrects an incorrect initial response to arrive at a final correct answer, this is still considered an error and is included in the analysis of incorrect responses.

Additionally, eye-tracking will be used to examine real-time insight into NPH patients’ language processing characteristics during vocabulary retrieval. Eye-tracking provides valuable insights into visual cognitive processes by recording where and how long a participant gazes, thus enabling a dynamic examination of cognitive processing and information processing (Ungrady et al., 2019; Wilkinson and Mitchell, 2014). Eye-tracking variables are typically categorized into time variables (e.g., fixation duration), spatial variables (e.g., saccade scanpath), or frequency variables (e.g., fixation count). Variables related to time and frequency are associated with cognitive processing, while spatial variables are related to visual perception abilities (Lai et al., 2013). Previous studies have demonstrated that older adults exhibit increased fixation durations during more challenging object-naming tasks, while individuals with cognitive impairments demonstrate slower eye movements and higher fixation frequencies compared to normal elderly individuals (Meyer et al., 2003). Lehtola et al. (2022) analyzed fixation and saccade-related metrics during the King-Devick test, a brief rapid number-naming assessment, among idiopathic NPH, AD, and cognitively unimpaired elderly individuals. The study revealed markedly shorter saccade durations in the AD group compared to the normal elderly group. In contrast, both the idiopathic NPH and AD groups exhibited significantly reduced saccade amplitudes compared to the normal elderly group. Given the scarcity of eye-tracking research related to NPH, this study will analyze various eye-tracking metrics during the lexical retrieval task, including fixation duration and count, saccade duration, count, amplitude, and scanpath.

Moreover, this study aims to analyze eye-tracking data for both correct and incorrect responses. While prior research has primarily focused on quantitative differences in correct responses, this study investigates qualitative differences by comparing the eye-tracking patterns between correct and incorrect responses, in addition to examining quantitative differences. In a study by Ungrady et al. (2019), eye movement patterns during an object-naming task with 200 images were compared between patients with primary progressive aphasia (PPA) and AD, analyzing both correct and incorrect responses. Incorrect responses were characterized by fewer fixations, increased gaze shifts, and reduced gaze movement speeds. Therefore, this study will investigate language processing in individuals with NPH by analyzing eye-tracking data associated with both correct and incorrect responses, extending beyond the binary outcome of success or failure in word retrieval.

While certain patterns in the temporal, frequency, and spatial variables of eye tracking data may be indicative of cognitive impairment, it is important to interpret these results in the context of the specific situation, task involved, and difficulty of the task faced by the individual. For example, in some cases, greater spatial dispersion may reflect a more thorough exploration of the environment, possibly indicating curiosity or engagement with the material. However, if eye tracking is abnormal in other situations, especially those in which the individual is able to focus or absorb information effectively, it may indicate a cognitive impairment or difficulty controlling attention that prevents the individual from focusing on a particular area. Therefore, to determine whether a particular eye tracking pattern is indicative of cognitive impairment, it’s important to consider the context of the task and the individual’s behavior. For this reason, we designed an eye-tracking experiment based on individual behavioral differences between correct and incorrect responses in the context of a task in which participants were asked to stare at a picture of a single object and name it in a fully attentive environment. If the NPH group shows different eye tracking times and frequencies compared to the control group on the word retrieval task, this would indicate abnormalities in cognitive processing in this group. And if the NPH group shows unusual values of the spatial variables of eye tracking in the same task, it would indicate a functional deficit in their visual perception. Furthermore, if there are differences in eye tracking variables between correct and incorrect responses, this can also be interpreted as a difference caused by cognitive challenges faced by individuals.

In summary, this study aims to investigate the real-time language processing of NPH and normal elderly groups by comparing eye-tracking metrics during a lexical retrieval task, analyzing both correct and incorrect responses. Specifically, this study will first compare lexical retrieval task performance and essential eye-tracking metrics between the groups. Second, the relationship between lexical retrieval performance and eye-tracking metrics will be analyzed. Third, it will categorize the subjects’ responses as correct or incorrect and compare eye-tracking metrics and responses between these two groups. Finally, heatmaps and scanpaths will be used to qualitatively and quantitatively compare eye-tracking data visualizations for correct and incorrect items. These attempts will shed light on the language processing abilities of patients with NPH, an understudied area in clinical language research, and inform future directions for language assessment and intervention in this similarly neglected population.

## 2 Materials and methods

### 2.1 Participants

A total of 26 subjects participated in this study: 14 patients over the age of 65 diagnosed with NPH (NPH group) and 12 individuals from the healthy elderly population (HE group). The selection criteria for the NPH group were as follows: (1) gradual onset of symptoms; (2) symptom duration of at least 3 to 6 months; (3) absence of other diseases that could explain the clinical symptoms or imaging findings; (4) evidence of ventricular enlargement; (5) presence of gait disturbance, cognitive impairment, and urinary dysfunction; (6) no increase in cerebrospinal fluid pressure based on diagnostic criteria (70–245 mmH_2_O); and (7) patients who had not yet undergone a shunt procedure. Participants in the NPH group were selected based on the above diagnostic criteria following a consensus between a neurosurgeon and a neurologist.

Participants in the HE group were selected based on the absence of sensory, neurological, or physical impairments and performance within the normal range on the Korean Mini-Mental State Examination-2 (K-MMSE2), specifically with a score at or above the 16th percentile. The mean age was 79.14 years (SD = 4.13) in the NPH group and 76.33 years (SD = 3.23) in the HE group, with no significant difference between the two groups (*U* = 50.000, *Z* = −1.761, *p* = 0.078). The mean years of education were 7.00 years (SD = 5.80) in the NPH group and 10.08 years (SD = 2.84) in the HE group. K-MMSE2 scores were 19.93 (SD = 4.98) in the NPH group and 26.58 (SD = 1.93) in the HE group. While there were no significant differences between the two groups in terms of gender (x^2^ = 0.000, *p* = 1.00) and years of education (*U* = 55.000, *Z* = −1.530, *p* = 0.126), significant disparities were observed in K-MMSE2 scores (*U* = 17.000, *Z* = −3.463, *p* = 0.001).

### 2.2 Lexical retrieval task (LRT)

In the lexical retrieval task (LRT), participants verbally named the object depicted in a presented picture. This task assessed lexical retrieval using the approach described by Dell et al. (1997). Since the primary objective of this task was to assess the underlying lexical retrieval process, only the first verbal response produced upon viewing the picture was scored. A response was classified as correct when the participant successfully named the depicted object. In cases where the participant failed to produce a response, no prompts or cues were provided, and the response was recorded as incorrect. Additionally, responses that were initially incorrect but subsequently corrected through self-correction were also classified as incorrect. Additionally, the error types of the responses were analyzed by categorizing them into semantic errors, formal errors, mixed errors, unrelated errors, and nonword errors, following the classification outlined in the previous study by Dell et al. (1997). These data were utilized for supplementary analysis to aid in the interpretation of the results; therefore, they were not separately presented as the main findings of this study.

The stimulus words for this task were selected from the word list developed by Kim et al. (2024).Stimuli were selected based on their frequency as common nouns prone to errors in semantic lemma selection due to the presence of multiple words within the same semantic category. Furthermore, the words were selected based on their potential for phonological complexity, which could induce errors in lexical retrieval. The specific selection criteria are as follows: The words for the LRT were selected based on previous research findings (Ryu, 2012), which indicate that individuals with cognitive impairment exhibit variations in error frequency depending on word frequency and syllable length. Jo (2002) investigated the frequency of Korean words, including proper nouns, particles, and verb endings, classifying words with a frequency below 15 as low-frequency and those with a frequency of 15 or higher as high-frequency. Based on this classification, the present study included 13 high-frequency words and 17 low-frequency words. In terms of syllabic complexity, the selected words comprised five two-syllable words, 13 three-syllable words, 11 four-syllable words, and one five-syllable word. To increase task difficulty, a greater proportion of three- and four-syllable words was included. Furthermore, the target words were systematically selected to ensure balanced representation across 15 semantic categories: land animals (2), insects (2), fruits (2), vehicles (2), household items (2), musical instruments (2), marine animals (1), wetland animals (1), vegetables (2), kitchen utensils (2), flowers (2), tools (4), artificial objects (3), additional kitchen utensils (2), and birds (2). The word list for the LRT is provided in Appendix 1.

### 2.3 Procedures

**Figure 1:**
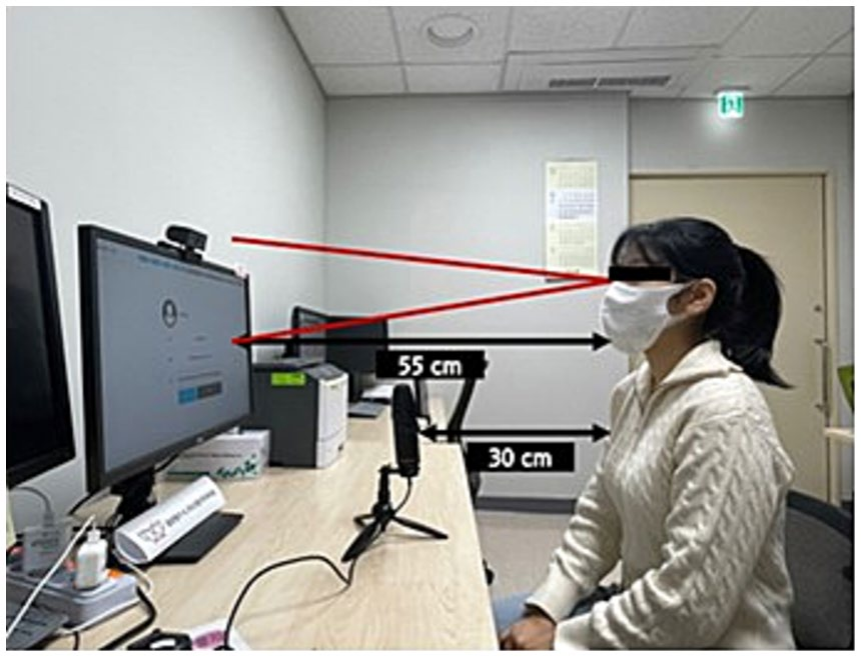
Experiment setup.

This study employed naming task software with eye-tracking functionality for experimental analyses. The software comprises two main modules: one for inputting examiner and subject information and another for implementing and analyzing the naming task. Participants who responded to the prompts from the lexical retrieval task module were automatically recorded and saved to Google Drive. Each participant completed the experiment individually in a quiet, controlled environment. Before the commencement of the task, an eye-tracking camera was mounted on top of the monitor, and a microphone was strategically positioned to record participant verbal responses. Participants were seated 55 cm from the monitor and 30 cm from the microphone (Figure 1). Before starting the task, participants received thorough instructions on the procedure and were asked to name the pictures as quickly and accurately as possible. Participants then completed a calibration process for the eye tracker, which required them to gaze at five sequentially presented dots on the screen. Participants proceeded to the task only after successfully completing the calibration process.

### 2.4 Eye-tracking apparatus and eye movement measures

#### 2.4.1 Eye-tracking apparatus

The experimental setup utilized a Dell E2422HS display featuring a 1,920 × 1,080 pixel resolution for stimulus presentation. Gaze tracking was accomplished using a Pengca Webcam 1080p, which captured eye movements at 30 frames per second. The actual data collection sampling rate was approximately 28.43 Hz, demonstrating minimal data loss and stable recording conditions throughout the experiment. A Samsung Galaxy Book2 (model NT550XEZ-A58A) served as the host computer, processing eye-tracking data and managing stimulus delivery to the Dell monitor. The eye-tracking software employed in this study was SeeSo (available at https://visual.camp/demo-archive/), developed by VisualCamp Co., Ltd. in Seoul, Korea. This software has a reported gaze-tracking accuracy of approximately 1.7°.

**Figure 2:**
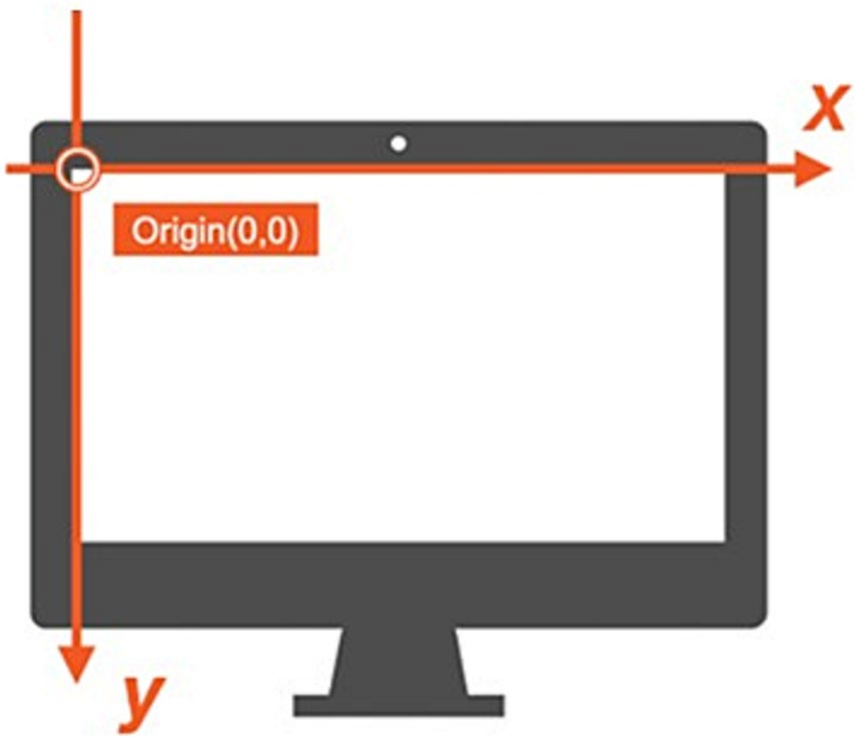
Gaze coordinates.

Before data collection, each participant underwent a calibration process using the five-point procedure within the SeeSo interface. Calibration involved fixating on five points: one at the center and four at the corners of the monitor. Calibration accuracy, assessed by successful fixation on each calibration point, ensured reliable gaze tracking throughout the experiment. Calibration was considered successful when the participant’s gaze remained within 1.7° of each target point for 5 s. This criterion was applied to all five calibration points, and the experiment proceeded only after successful calibration at all points. If calibration failed at any point, the entire process was repeated until successful calibration was achieved. Notably, the gaze coordinate system originated at the top-left corner of the screen (0,0), as shown in Figure 2. This coordinate system used pixel-based measurements, where horizontal coordinates (x-axis) increased from left to right, and vertical coordinates (y-axis) increased from top to bottom across the 1,920 × 1,080 pixel display. This pixel-based coordinate system enabled precise tracking of eye movements with spatial resolution at the pixel level, facilitating detailed analysis of fixation locations and saccade trajectories during the experimental tasks. The eye-tracking data included x- and y-coordinates for each gaze point, along with timestamp information. Gaze points were classified as either fixations or saccades during preprocessing. A fixation was defined as when the participant’s gaze remained stationary on a specific area of the screen for at least 70 ms. Rapid eye movements between fixation points were classified as saccades, specifically when the gaze movement velocity between two consecutive fixation points exceeded 30°/s. The SeeSo software’s velocity-based algorithm was used for this classification process. When a gaze point belonged to a fixation, all gaze coordinates within that fixation period (from the first to the last fixation-classified gaze point) were transformed to match the initial fixation point’s coordinates. This method ensured consistency in fixation location analysis. In contrast, gaze points classified as saccades, representing transitional data, were excluded from the analysis.

**Figure 3:**
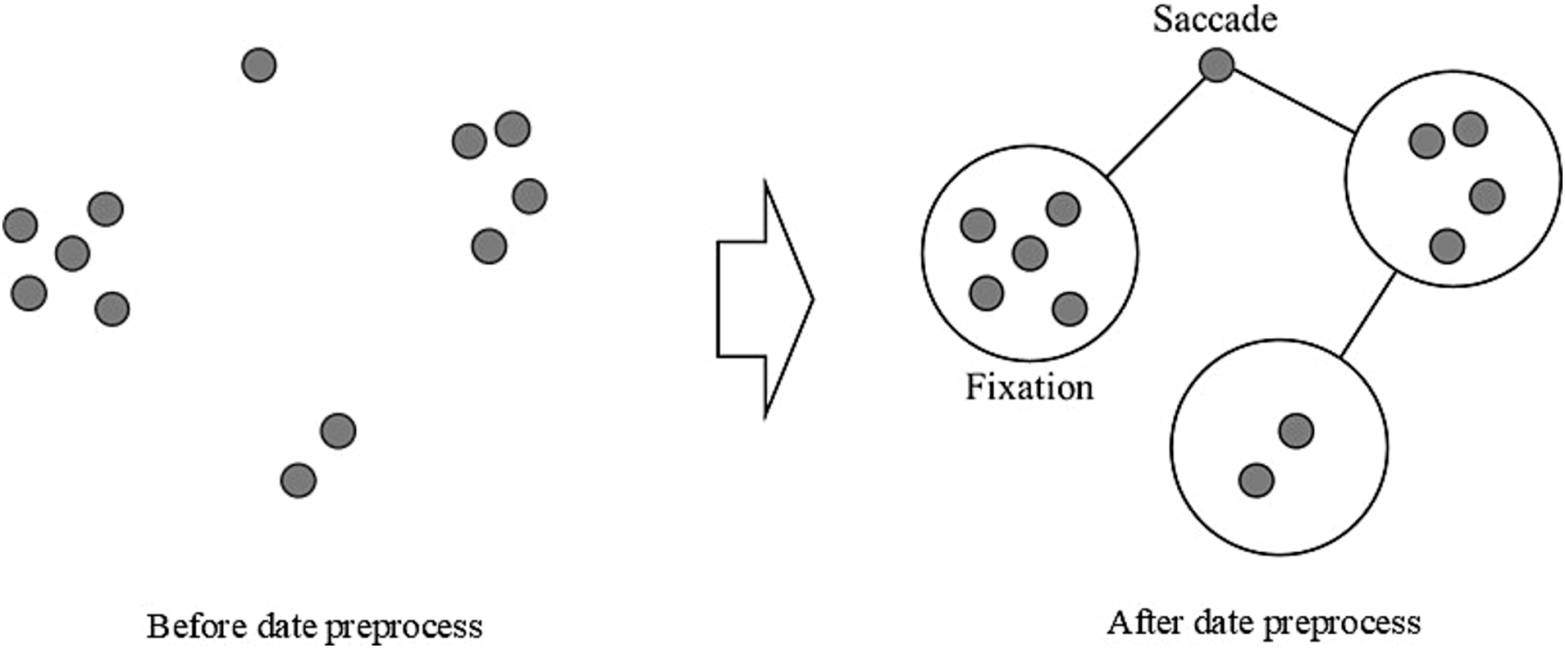
Gaze data preprocessing.

Figure 3 illustrates how a one-time point identified as a saccade was considered an intermediate process and excluded, allowing the determination of gaze shifts between fixations. This preprocessing methodology improved the accuracy of fixation and saccade measurement by eliminating transitional noise from the data. This precise coordinate system, coupled with the high-resolution display and accurate eye-tracking hardware, enabled detailed analysis of fixation points, saccade patterns, and areas of visual interest within the stimuli. We analyzed a specific Area of Interest (AOI) covering the central image display region with dimensions of 1,483 × 707 pixels, rather than the entire display area. This focused approach ensured our eye-tracking metrics reflected visual processing of the stimulus images, excluding extraneous eye movements toward peripheral screen regions. All gaze data collected outside this defined AOI were excluded from the analysis. This decision was made to ensure that our analysis reflected only the visual processing of the stimulus images, eliminating the influence of extraneous eye movements toward peripheral screen regions. Thus, all fixation and saccade metrics reported in this study were calculated only within this defined AOI.

#### 2.4.2 Eye movement measures

Our investigation utilized six key eye-tracking parameters to analyze visual behavior during a picture-naming task. Eye-tracking metrics in Table 1 are widely used to measure cognitive load and visual attention in various neurological conditions, providing insights into how cognitive impairments affect visual processing strategies (Hutchings et al., 2018; MacAskill et al., 2012; Wang et al., 2014).

**Table 1:** Summary of eye-tracking metrics used in the study.

Feature category	Feature	Description
Fixation	Fixation Duration (FD)	Total time gaze remains stable on a specific area
	Fixation Count (FC)	Number of times fixations occur during the task
Saccade	Saccade Count (SC)	Number of rapid eye movements between fixations
	Saccade Duration (SD)	Cumulative time spent in saccadic movements
	Saccade Scanpath Total (SST)	Total distance traveled by eyes during saccades
	Saccade Amplitude Total (SAT)	Sum of angular displacements from initial to final positions of saccades

Fixation Duration (FD) is calculated as $FD=\sum \left(t_{\mathrm{end}}-t_{\mathrm{start}}\right)$ for all fixations where ( $t_{\mathrm{end}}$ − $t_{\mathrm{start}}$ ) ≥ 70 ms. Here, $t_{\mathrm{start}}$ represents the time at which a fixation begins and $t_{\mathrm{start}}$ indicates the time at which it concludes. Fixation is when the eyes remain relatively stationary, concentrating on a certain point. We consider a fixation valid only if it lasts at least 70 milliseconds. This metric explains the cumulative time participants spend focusing on specific image areas. In the realm of cognitive impairment, variations in fixation duration could indicate alterations in information processing efficiency. For instance, in Alzheimer’s disease, longer fixation durations are associated with impairments in visual search and attention (Pereira et al., 2020). Within the context of NPH, alterations in fixation duration may signify the cognitive delays and attentional deficits observed in this condition.

Fixation Count (FC) is the total number of fixations recorded throughout the task period. Every time the eye pauses to concentrate on a point, it is counted as a single fixation. This metric assists in quantifying the frequency with which participants focus on different sections of the images. In neurodegenerative disorders, fluctuations in fixation count may indicate alterations in visual search patterns or attentional control. Interestingly, recent research by Hutchings et al. (2018) on behavioral-variant frontotemporal dementia patients revealed increased fixations to the eyes of emotional faces compared to healthy controls, suggesting that some cognitive disorders may result in atypical visual attention patterns. For NPH patients, alterations in fixation counts could suggest changes in visual search efficiency or attentional control, which are cognitive domains frequently impacted in this condition.

Saccade Count (SC) represents the total number of rapid eye movements between fixations. A saccade is characterized by the eye quickly moving from one fixation point to another. This measurement supplies data on the frequency of gaze shifts during the task. In Parkinson’s disease, alterations in saccadic eye movements have been linked to cognitive decline and executive dysfunction (Chan et al., 2005). For patients with cognitive impairments, variations in saccade count could signal differences in the capacity to shift attention or explore visual scenes efficiently. In NPH, fluctuations in saccade count could potentially reflect alterations in the capability to effectively shift attention or explore visual scenes, which may be compromised due to the cognitive deficits associated with the condition.

Saccade Duration (SD) is calculated as $SD=\sum \left(s_{\mathrm{end}}-s_{\mathrm{start}}\right)$ for all saccades. Here, $s_{\mathrm{start}}$ is the time when a saccade begins, and $s_{\mathrm{end}}$ is the time when it concludes. This metric can offer insights into the efficacy of visual exploration and the capacity to quickly shift attention between various areas of interest. In the realm of NPH, where cognitive impairment is a prominent characteristic, we could expect to see alterations in saccade duration that reflect underlying cognitive changes. For NPH patients, variations in total saccade duration may signify alterations in the speed and efficiency of visual exploration, potentially reflecting the cognitive slowing characteristic of this condition.

Saccade Scanpath Total (SST) is computed as $SST=\sum \sqrt{{\left(x_{\mathrm{end}}-x_{\mathrm{start}}\right)}^{2}+{\left(y_{\mathrm{end}}-y_{\mathrm{start}}\right)}^{2}}$ for all saccades. In this equation, ( $x_{\mathrm{start}}$ , $y_{\mathrm{start}}$ ) represents the coordinates of the starting point of a saccade, and ( $x_{\mathrm{end}}$ , $y_{\mathrm{end}}$ ) represents the coordinates of the ending point. This metric can uncover alterations in the scope and pattern of visual exploration, which might be modified in conditions impacting cognitive function. For instance, Boz et al. (2023) identified that Alzheimer’s disease (AD) patients display inefficiencies in visual scanning of their surroundings, directing their attention towards non-relevant aspects of scenes. In NPH, modifications in scanpath patterns could indicate alterations in visual search strategies and spatial cognition, which may be influenced by the cognitive impairments linked with this condition.

Saccade Amplitude Total (SAT) is calculated as $SAT=\sum \tan^{-1}\left[\left(y_{\mathrm{end}}-y_{\mathrm{start}}\right)/\left(x_{\mathrm{end}}-x_{\mathrm{start}}\right)\right]$ for all saccades. This metric offers insight into the magnitude of gaze shifts, potentially indicating changes in the ability to make large eye movements or explore wider areas of the visual field. In neurodegenerative conditions, reduced saccade amplitudes could indicate challenges in planning or performing larger eye movements. For NPH patients, changes in saccade amplitudes could indicate challenges in planning or executing larger eye movements, potentially stemming from the cognitive and motor impairments linked with the condition.

Also, we implemented a grid-based quantitative analysis approach. Each stimulus image was divided into a 10 × 10 grid (creating 100 equal cells), and fixation distributions across this grid structure were analyzed. We calculated multiple metrics to quantify different aspects of visual scanning behavior:

1. *Grid Entropy*: This metric measures the randomness or uncertainty in the distribution of fixations across the grid cells. Calculated using Shannon’s entropy formula: $H=-\sum \left(p_{i}\times \log p_{i}\right)$ where $p_{i}$ is the probability of a fixation occurring in cell i . Higher values indicate more dispersed attention patterns with fixations distributed more evenly across the grid.
2. *Spatial Dispersion*: Quantifies the average Euclidean distance between consecutive fixations, reflecting how widely distributed the visual exploration was. Larger values indicate greater distances between fixations and more extensive scanning.
3. *Grid Occupancy*: The proportion of grid cells that contained at least one fixation (range: 0–1). Higher values indicate that participants explored a larger portion of the stimulus area.
4. *Transition Density*: The ratio of unique transitions between grid cells to the total possible transitions. This metric reflects the complexity of visual search patterns, with higher values indicating more varied transitions between areas of the stimulus.
5. *Nearest Neighbor Index (NNI)*: Measures the degree to which fixations are clustered or dispersed. Values below 1 indicate clustering, while values above 1 suggest dispersion. This metric helps quantify the spatial organization of fixations.

For each analysis, we distinguished between total fixations and “inside fixations” (those that fell within the defined Area of Interest), to ensure accurate assessment of participants’ engagement with the stimulus images.

### 2.5 Statistics

Statistical analyses were conducted utilizing IBM SPSS, version 27.0. Given that the assumption of normality was violated, a Mann–Whitney U test was performed to compare LRT score and eye-tracking metric scores between the NPH and HE groups. Pearson’s correlation analysis was conducted to assess the relationships between LRT score and each eye-tracking index. A two-way mixed ANOVA was performed to examine the significance of the eye-tracking metrics based on response type (correct/incorrect) between the two groups, followed by Bonferroni-corrected *post hoc* comparisons. Additionally, a post-hoc power analysis was performed using G*Power 3.1 to ensure the study had adequate statistical power (Appendix 2).

## 3 Results

### 3.1 LRT score

The Mann–Whitney U test results revealed that the NPH group demonstrated significantly lower performance than the HE group (*U* = 2.500, *p* = 0.000) in LRT. The NPH group had an average LRT score of 52.14 (SD = 16.88), while the HE group scored significantly higher, with an average of 84.17 (SD = 7.26). A post-hoc power analysis demonstrated that the statistical power exceeded 90%, indicating sufficient sample size to detect significant effects.

### 3.2 Eye-tracking metrics

All participant responses were analyzed using eye-tracking metrics. As shown in Table 2, the Mann–Whitney U test revealed significant differences between the NPH and HE groups for all eye-tracking metrics. Specifically, the NPH group demonstrated significantly longer fixation durations (*U* = 19.000, *Z* = −3.343, *p* = 0.001) and saccade durations (*U* = 20.000, *Z* = −3.806, *p* = 0.001). Additionally, the NPH group exhibited significantly higher fixation counts (*U* = 2.000, *Z* = −3.292, *p* = 0.001) and saccade counts (*U* = 20.000, *Z* = −3.292, *p* = 0.001) than the HE group. In the NPH group, the total saccade scanpath was also significantly longer (*U* = 13.000, *Z* = −3.652, *p* = 0.000) and the total saccade amplitude was greater (*U* = 15.000, *Z* = −3.549, *p* = 0.000). A post-hoc power analysis indicated that the statistical power exceeded 90%, confirming the adequacy of the sample size for detecting significant effects.

**Table 2:** Mann–Whitney U test results of eye-tracking metrics between NPH group and HE group.

Feature	NPH (*n* = 14)	HE (*n* = 12)	*U*	*p*
	Mean (SD)	Mean (SD)		
FC	22.63 (8.31)	12.31 (3.06)	2.000	0.001
FD	6139.76 (2239.67)	3263.67 (889.09)	19.000	0.001
SC	21.65 (8.30)	11.31 (3.06)	20.000	0.001
SD	4143.48 (1489.44)	2018.58 (612.86)	10.000	0.000
SST	6064.02 (3075.49)	2617.30 (1067.94)	13.000	0.000
SAT	666.74 (278.87)	311.28 (101.26)	15.000	0.000

NPH, Patients with normal pressure hydrocephalus; HE, Health elderly controls; FC, fixation count; FD, fixation duration (ms); SC, saccade count; SD, saccade duration (ms); SST, saccade scanpath total (pixel); SAT, saccade amplitude total (degree).

### 3.3 Relationship between lexical retrieval task score and eye-tracking metrics

The correlation analysis results revealed that lexical retrieval task scores were negatively correlated with FC (*r =* −0.569, *p = 0*.002), FD (*r =* −0.566, *p = 0*.003), SC (*r =* −0.569, *p = 0*.002), SD (*r =* −0.554, *p = 0*.003), SST (*r =* −0.488, *p = 0*.011), and SAT (*r =* −0.462, *p = 0*.017). Scatter plots of correlation analysis results are provided in Appendix 3. Post-hoc power analysis revealed these sample sizes provided over 80% power except for SAT (>74%) and SST (>68%) for detecting a significant relationship.

### 3.4 Eye-tracking metrics based on correct and incorrect responses

Table 3 displays descriptive statistics for the eye-tracking metrics, categorized by response accuracy. Overall, the NPH group exhibited significantly higher counts and longer durations across all eye-tracking metrics compared to the HE group. Furthermore, both groups exhibited higher frequencies and longer durations for these metrics during incorrect responses compared to correct ones.

**Table 3:** Descriptive statistics of eye-tracking metrics for both correct and incorrect responses.

Feature	NPH (*n* = 14)		HE (*n* = 12)	
	Correct	Incorrect	Correct	Incorrect
	Mean (SD)	Mean (SD)	Mean (SD)	Mean (SD)
FC	14.05 (4.18)	34.23 (14.84)	9.94 (2.08)	22.26 (8.90)
FD	3925.58 (1352.17)	9216.46 (4324.67)	2634.16 (640.07)	5663.05 (2401.56)
SC	13.08 (4.14)	33.44 (14.62)	8.94 (2.08)	21.26 (8.90)
SD	2249.65 (634.63)	6425.71 (2080.53)	1658.81 (490.01)	4152.57 (1324.87)
SST	3599.13 (1695.89)	9484.76 (5620.43)	2076.57 (753.80)	5236.22 (3013.62)
SAT	415.81 (135.81)	978.90 (395.64)	241.02 (76.47)	713.40 (418.35)

NPH, Patients with normal pressure hydrocephalus; HE, Health elderly controls; FC, fixation count; FD, fixation duration (ms); SC, saccade count; SD, saccade duration (ms); SST, saccade scanpath total (pixel); SAT, saccade amplitude total (degree).

FC tended to be higher during incorrect responses in both groups, with a larger increase observed in the NPH group (M = 34.23, SD = 14.84) compared to the HE group (M = 22.26, SD = 8.90). Similarly, FD increased during incorrect responses, with the NPH group exhibiting a greater increase (M = 9216.46 ms vs. 3925.58 ms for correct responses) than the HE group (M = 5663.05 ms vs. 2634.16 ms). Examining the saccade-based metrics, both groups exhibited a higher SC during incorrect responses, with a larger increase in the NPH group (M = 33.44, SD = 14.62) compared to the HE group (M = 21.26, SD = 8.90). Similarly, SD increased during incorrect responses, with the NPH group demonstrating a nearly threefold increase (M = 6425.71 ms vs. 2249.65 ms for correct responses), whereas the HE group exhibited a comparatively smaller increase (M = 4152.57 ms vs. 1658.81 ms). Both SST and SAT tended to be higher in incorrect responses across both groups, with a greater increase observed in the NPH group. The NPH group’s SST increased from 3599.13 pixels (correct) to 9484.76 pixels (incorrect), whereas the HE group showed a more moderate increase (2076.57 pixels to 5236.22 pixels). Similarly, SAT increased nearly twofold in both groups, with the NPH group exhibiting a greater absolute increase (M = 978.90 vs. 415.81 for correct responses) compared to the HE group (M = 713.40 vs. 241.02 for correct responses).

**Figure 4:**
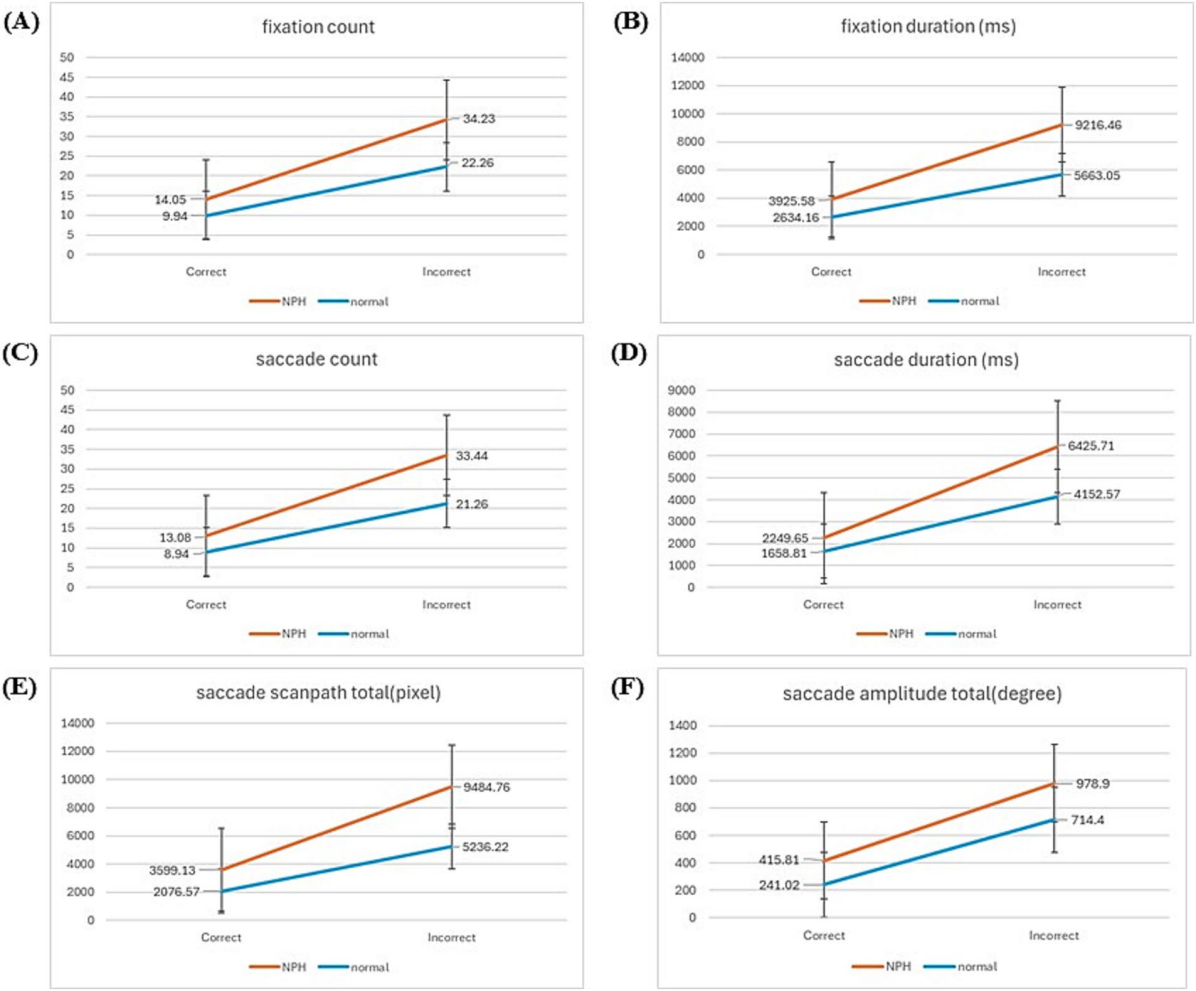
Graph of the results of two-way mixed ANOVA. **(A)** Fixation count. **(B)** Fixation duration. **(C)** Saccade count. **(D)** Saccade duration. **(E)** Saccade scanpath total. **(F)** Saccade amplitude total.

A two-way mixed ANOVA (group x response: correct, incorrect) revealed significant main effects of both group and response accuracy across all comparisons (Figure 4). The NPH group exhibited longer FD (*F*_[1,24]_ = 8.281, *p* = 0.008), SD (*F*_[1, 24]_ = 13.600, *p* = 0.001), and SST (*F*_[1,24]_ = 6.576, *p* = 0.017) than the HE group, irrespective of response type, along with a higher frequency of FCs (*F*_[1,24]_ = 7.534, *p* = 0.011), SCs (*F*_[1,24]_ = 7.956, *p* = 0.009) and greater SATs (*F*_[1, 24]_ = 6.109, *p* = 0.021). Significant within-group main effects were observed across all eye-tracking metrics, including FC (*F*_[1,24]_ = 60.922, *p* = 0.000), FD (*F*_[1,24]_ = 46.643, *p* = 0.000), SC (*F*_[1,24]_ = 62.539, *p* = 0.000), SD (*F*_[1,24]_ = 94.014, *p* = 0.000), SST (*F*_[1,24]_ = 39.284, *p* = 0.000) and SAT (*F*_[1,24]_ = 46.093, *p* = 0.000), indicating that, irrespective of group differences, both groups exhibited increased values across all eye-tracking measures during incorrect responses compared to correct responses. However, a significant interaction effect between group and response accuracy was observed only for SD (*F*_[1,24]_ = 5.981, *p* = 0.022), indicating that the increase in saccade duration from correct to incorrect responses was significantly greater in the NPH group than in the HE group. Post-hoc power analysis demonstrated that the statistical power exceeded 80%, confirming the sample size’s adequacy for detecting significant effects.

### 3.5 Visualization of eye-tracking data for correct and incorrect response items

#### 3.5.1 Qualitative analysis using heatmaps and scanpaths

Two items (“motorcycle,” “eggplant”) had a correct response rate of over 90%, and two items (“xylophone,” “escalator”) with an incorrect response rate of over 60% in both groups were selected.

**Figure 5:**
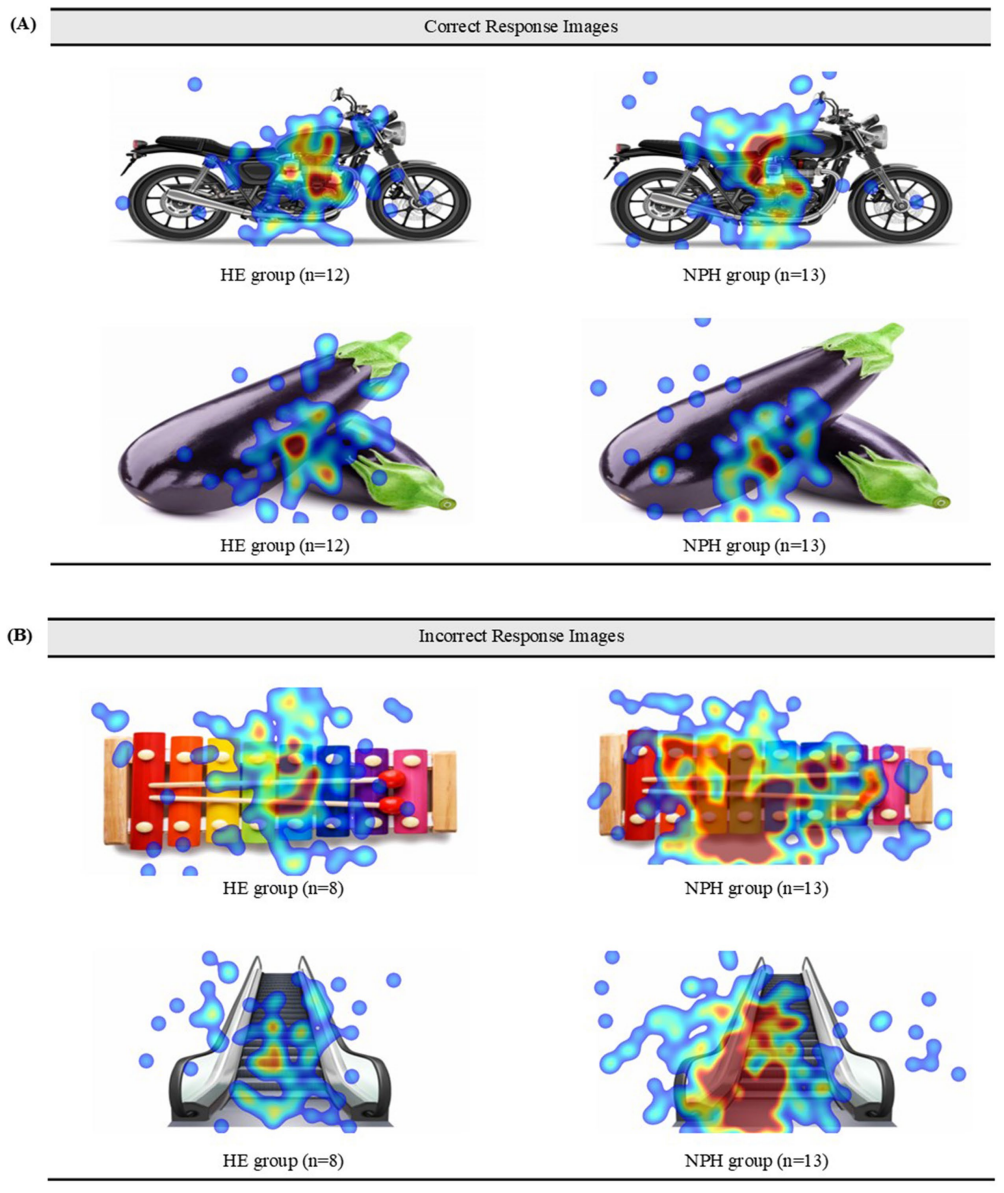
**(A)** Heatmaps of the correct items for both groups. **(B)** Heatmaps of the incorrect items for both groups.

Heatmaps and scanpaths were used to visualize and compare fixations and saccades between the two groups for correctly and incorrectly answered items. First, heatmaps for four images (two corresponding to a correct item and two to an incorrect item) are displayed in Figures 5A,B. For correctly identified items, the NPH group exhibited a more dispersed gaze pattern than the HE group, frequently fixating on areas beyond the image. This difference was particularly pronounced for the incorrect response items. The HE group focused centrally on the images, while the NPH group exhibited a more dispersed gaze pattern, particularly in the image of the escalator.

Scanpaths for both groups are presented in Appendix 4, using the “motorcycle” as a representative of the correct items and the “xylophone” as a representative of the incorrect items. Upon comparing the overall characteristics of gaze movement paths between the two groups, the HE group exhibited a higher complexity in gaze paths in responding to the incorrect item compared to the correct item. In contrast, the NPH group demonstrated more diverse gaze movement paths in the correct items compared to the HE group, with even greater complexity observed in the incorrect item. Individual variability, particularly within the NPH group, should also be considered. Some NPH participants who demonstrated a simple gaze path in response to the incorrect item also exhibited fewer gaze movements in response to the incorrect item. For instance, patient 04 (P04) displayed a deviant gaze path despite successfully naming a “motorcycle” and a very simple scanpath despite misidentifying the “xylophone.”

#### 3.5.2 Quantitative analysis using a grid-based approach

To statistically validate our qualitative observations of eye movement patterns (heatmaps and scanpaths), we additionally employed a grid-based quantitative analysis approach (Appendix 5). Table 4 presents the results of our grid-based analysis for both correct (“motorcycle,” “eggplant”) and incorrect (“xylophone,” “escalator”) response items across NPH and HE groups. The NPH group consistently exhibited higher grid entropy values for incorrect response items (xylophone: 5.58; escalator: 5.24) than the HE group (xylophone: 5.45; escalator: 5.00). Incorrect items (xylophone: 5.20; escalator: 4.30) elicited substantially greater spatial dispersion in the NPH group compared to the HE group (xylophone: 2.20; escalator: 1.35). This suggests that when NPH patients struggled with word retrieval, their eye movements traversed significantly greater distances between fixations. When responding incorrectly, the NPH group exhibited greater visual exploration of the stimulus area, demonstrated by higher grid occupancy values (xylophone: 0.72; escalator: 0.60) than the HE group (xylophone: 0.59; escalator: 0.38). This observation is consistent with the broader finding that NPH patients exhibited greater gaze dispersion during unsuccessful naming attempts.

**Table 4:** Grid-based analysis of eye movement patterns for correct and incorrect response items.

Response type	Item	Group	Inside fixations	Grid entropy	Spatial dispersion	Grid occupancy	Transition density	NNI
Incorrect	Xylophone	NPH	400	5.58	5.20	0.72	0.92	0.87
		HE	157	5.45	2.20	0.59	0.94	0.82
	Escalator	NPH	266	5.24	4.30	0.60	0.92	0.77
		HE	83	5.00	1.35	0.38	0.93	0.74
Correct	Motorcycle	NPH	110	4.95	1.88	0.40	0.93	0.79
		HE	96	4.86	1.77	0.39	0.95	0.75
	Eggplant	NPH	82	4.62	1.66	0.33	0.96	0.69
		HE	73	4.57	1.51	0.29	0.90	0.69

NPH, Patients with normal pressure hydrocephalus; HE, Health elderly controls. Grid entropy measures fixation distribution randomness; Spatial dispersion measures average distance between fixations; Grid occupancy represents proportion of grid cells with fixations; Transition density measures complexity of transitions between cells; NNI (Nearest Neighbor Index) measures fixation clustering tendency.

Interestingly, for correct response items, group differences were less pronounced across all metrics. Grid entropy values were similar between NPH and HE groups for both “motorcycle” (NPH: 4.95; HE: 4.86) and “eggplant” (NPH: 4.62; HE: 4.57). Grid occupancy was also comparable (motorcycle—NPH: 0.40, HE: 0.39; eggplant—NPH: 0.33, HE: 0.29).

## 4 Discussion

This study utilized eye-tracking techniques to investigate the language processing characteristics of NPH patients. The NPH group displayed significantly lower performance in lexical retrieval compared to the HE group and showed higher counts and longer durations in both fixation and saccade metrics. Lexical retrieval scores and eye-tracking metrics were negatively correlated, with coefficients primarily ranging from 0.40 to 0.60, indicating a moderate correlation. Analysis of eye movements during correct and incorrect responses indicated the NPH group exhibited more frequency and longer duration of eye-tracking metrics compared to the HE group. Additionally, both groups demonstrated increased frequency and longer duration of these metrics in incorrect responses compared to correct responses. Although the interaction effect between group and response was not significant for most metrics, a significant interaction was observed for saccade duration. Both qualitative and quantitative analyses using heatmaps and scanpaths further confirmed more pronounced differences between the two groups for incorrect responses.

### 4.1 Comparison of lexical retrieval and eye movement between NPH and HE groups

The NPH group demonstrated greater lexical retrieval difficulties than the HE group, consistent with previous research on language impairments in NPH patients (Cho et al., 2024; Chung, 2018; Kim et al., 2024). This finding may reflect the significantly lower cognitive function of the NPH group compared to the HE group, suggesting that language deficits in the NPH group are secondary to cognitive impairment. However, the significant difference in lexical retrieval performance, despite the NPH group not exhibiting severe cognitive impairment (K-MMSE = 20.33), warrants further interpretation in conjunction with the eye movement findings.

The NPH group exhibited significantly longer fixation durations (*U* = 19.000, *Z* = −3.343, *p* = 0.001) and higher fixation count (*U* = 2.000, *Z* = −3.292, *p* = 0.001) than the HE group. Fixation duration represents the time spent focused on a single point before the gaze shifts, while fixation count represents the number of times the eyes fixate on different points or objects. Thus, prolonged fixation durations suggest a potential impairment in the ability of NPH patients to shift their attention from one point to another. Frequent fixation counts indicate that, while viewing images during the lexical retrieval task, the NPH group may have paused more frequently, implying less efficient visual stimulus scanning than the HE group. Moreover, the NPH group exhibited longer saccade durations (*U* = 20.000, *Z* = −3.806, *p* = 0.001) and a greater number of saccade counts (*U* = 20.000, *Z* = −3.292, *p* = 0.001) than HE group. Prolonged saccade durations indicate slower eye movements, while higher saccade counts indicate more frequent eye movements. This suggests that the NPH group, requiring more eye movements to complete the task, may process visual information in a more fragmented manner. Since the frontal lobe is involved in attention shifting and the basal ganglia in ocular motor control (Hikosaka et al., 2000), ventricular enlargement in NPH patients may exert pressure on these structures, potentially impairing visual information processing and consequently affecting lexical retrieval.

These findings align with previous research demonstrating the secondary effects of cognitive processing impairments on language activities in NPH patients (Cho et al., 2024; Kim et al., 2024; Whitehouse et al., 1993). The findings of this study suggest that lexical retrieval difficulties in NPH patients may stem from cognitive impairment and weakened visual processing abilities. In contrast, the research conducted by Lehtola et al. (2022), employing eye-tracking techniques, revealed varying results, where the NPH group exhibited shorter saccade durations than the AD group, and both the NPH and AD groups displayed reduced amplitudes compared to the control group. This discrepancy may stem from differing task differences, as the prior study employed number-naming tasks, whereas the current study analyzed eye movements during picture-naming tasks.

Another finding is that, as aging progresses, oculomotor ability tends to decrease, increasing both the counts and durations of eye movements. Moreover, oculomotor abilities are significantly more diminished in patients with neurocognitive disorders like PD and AD compared to those experiencing a typical age-related decline (Lehtola et al., 2022; Peltsch et al., 2011; Rösler et al., 2000). Significantly diminished oculomotor abilities in NPH patients compared to the HE group suggest that these differences may reflect the combined effects of age-related decline in visual processing speed and neurological damage. Consequently, the increased cognitive and linguistic demands experienced by NPH patients, similar to those observed in other neurocognitive disorders, highlight the importance of proactive cognitive and language rehabilitation interventions.

### 4.2 Metric differences in eye movement based on correct and incorrect responses

Both groups exhibited increased in fixation-related metrics (fixation count and fixation duration) and saccade-related metrics (saccade count, saccade duration, scanpath length and saccade amplitude) during incorrect responses compared to correct responses. These findings align with previous research showing longer fixation durations for more complex and less familiar words during naming tasks in neurotypical groups (Meyer et al., 2003). The NPH group showed a significantly greater increase in fixation and saccade indices for incorrectly named words compared to the HE group. Specifically, they exhibited longer fixation, saccade durations, and increased fixations and saccade counts. Further analysis of the types of incorrect answers demonstrated that the NPH group exhibited the highest occurrence of semantic errors (e.g., tiger → lion). In this study, participants were presented with a visual stimulus and asked to identify the depicted object, requiring the participants to go through the visual object recognition, semantic processing, lexical access, and phonological production stages (Goodglass and Wingfield, 1997). Therefore, it can be inferred that impairments in visual information processing, beginning with object recognition, may significantly contribute to language processing difficulties in NPH by obstructing successful word retrieval at the semantic level. A comprehensive understanding of visual and language processing difficulties in the NPH group requires analyzing both correct and incorrect responses.

Contrary to prior research, which found that individuals with PD and PPA exhibited lower fixation counts and higher saccade frequencies during incorrect responses on a naming task (Ungrady et al., 2019), the NPH group exhibited more fixations during such responses. This discrepancy suggests distinct visual processing characteristics in individuals with NPH, contrasting with those observed in individuals with PD or PPA. Individuals with PD may experience “fixation itself” difficulties, while the NPH group appears to encounter challenges associated with the “inefficiency of fixation.” Given the overlap in primary symptoms between PD and NPH, differential diagnosis is crucial (Kim et al., 2024; Picascia et al., 2019), and differences in fixation metrics may serve as a potential biomarker for this purpose.

Notably, among these results, that an interaction effect was observed for the saccade duration (SD) metric. This suggests that there is no difference in SD between the two groups in correct responses, indicating the NPH group is not as slow as the HE group, at least in terms of eye movement speed, when it comes to successful lexical retrieval. The visualization analysis presented next further clarifies this convergence between the two groups for correct responses.

### 4.3 Visualization differences in eye movement in relation to correct and incorrect items

This study aimed to visualize real-time visual processing using heatmaps and scanpaths for correct and incorrect items. Notably, for the correct items, fixations were concentrated predominantly on the center of the images in both groups. This pattern varied, however, for incorrect items. The HE group’s fixations were also concentrated in the center, similar to those observed with correct items, while the NPH group’s fixations were more dispersed toward peripheral areas. This suggests that when the NPH group correctly recognizes the name of an object, there is no qualitative disparity in gaze fixation compared to the HE group. However, when encountering unnamed objects, their gaze processing becomes inefficient in tracking errors. Previous studies have shown that subcortical lesions can disrupt connections between brain regions, impairing error monitoring (Mandal et al., 2020). Inefficient visual search patterns may have contributed to impaired error monitoring in the NPH group.

Scanpath representations revealed that both groups exhibited more complex eye movement trajectories during incorrect items. However, variations in the pattern and complexity of eye movements were apparent between the two groups. Compared to the HE group, the NPH group exhibited a higher frequency of eye movements deviating from the central area of the image and presented more complex scanpath. The scanpath, encompassing both fixations and saccades, reflects eye movement trajectories. Longer scanpath may indicate increased cognitive effort during word retrieval or greater uncertainty in visual processing (Kennard, 2002). Conversely, as illustrated in P04 of Appendix 2, some individuals with NPH exhibited relatively straightforward scanpaths for correct and incorrect items. After evaluating their naming performance, these individuals were found to have considerably lower performance within the NPH group. This finding suggests that although “simple” scanpath typically indicate “efficient” visual processing, in cases of incorrect responses, they may reflect “inactive” processing and unsuccessful word retrieval.

A further grid-based quantitative analysis result supports these findings, revealing an association between successful lexical retrieval and greater similarity in eye movement patterns between the groups. Quantitative analysis confirmed that incorrect responses in the NPH group were associated with more dispersed, extensive, and less efficient visual exploration patterns compared to the HE group, while successful lexical retrieval in NPH patients correlated with more normalized visual processing patterns.

## 5 Conclusion

In conclusion, this study demonstrated that NPH patients had different eye tracking frequency, time, and spatial dispersion from the control group, which confirmed the cognitive impairment and visual perception deficits of these patients. This abnormality was more pronounced in incorrect responses, so it was also confirmed that the cognitive burden increases as the difficulty of the task increases, which is ultimately reflected in abnormal eye tracking indicators. Therefore, based on these findings, the following conclusions and recommendations are proposed. First, NPH patients demonstrate impaired lexical retrieval during picture naming. To effectively assess these lexical retrieval deficits, multi-syllable and low-frequency words, similar to those used in this study. Second, lexical retrieval performance may also be influenced by eye-tracking efficiency. Individuals with similar naming scores demonstrate a correlation between efficient eye-tracking metrics and better naming tasks. Therefore, analyzing gaze efficiency may offer a more sensitive indicator of lexical retrieval deficits in NPH patients. Third, the most significant difference between healthy elderly individuals and NPH patients is that NPH patients exhibit inefficient gaze processing during incorrect word retrieval. Individuals with NPH exhibit prolonged fixation times and more frequent saccades to search for word names, yet they ultimately fail. Patients with naming difficulties and atypical eye-tracking patterns may benefit from interventions that incorporate intermediate steps to simplify and normalize gaze patterns before targeting accurate naming. Fourth, patients who exhibit an unusually straightforward gaze path despite unsuccessful lexical retrieval may represent those with the most severe deficits. These individuals may benefit from interventions that promote active visual exploration strategies prior to initiating lexical retrieval training.

This study used eye movement analysis to enhance NPH patients’ understanding of the real-time language processing characteristics of NPH patients. By analyzing eye movements in instances of failed word retrieval, the study revealed specific visual information processing patterns inherent to the NPH group, which were not observed during correct responses. This study highlights the previously underexplored language impairments in individuals with NPH through real-time visual processing analysis and emphasizes the importance of language intervention for this population.

A key limitation of this study was the small sample size. Future research should address this by including a larger cohort to enhance the reliability of findings. Additionally, future studies should implement comparative analyses between the NPH group and other groups, such as those with PD or AD, where differential diagnosis poses challenges. Eye-tracking analysis may offer potential biomarkers for differentiating among these groups. Furthermore, since NPH is categorized as a reversible form of dementia that may improve after surgery, future studies should investigate how word retrieval and visual processing abilities change post-operatively.

## Data Availability

The raw data supporting the conclusions of this article will be made available by the authors, without undue reservation.
