# Supplementary material for: # Language processing characteristics in normal pressure hydrocephalus: insights from eye-tracking analysis of incorrect responses
Source: Front Aging Neurosci. 2025 Apr 23;17:1527962. doi: 10.3389/fnagi.2025.1527962 (PMC12055843; doi:10.3389/fnagi.2025.1527962)

Supplementary Material

# Supplementary Figures Tables and appendix

## Supplementary Appendix

**Supplementary Appendix 1.** Word list for the lexical retrieval task


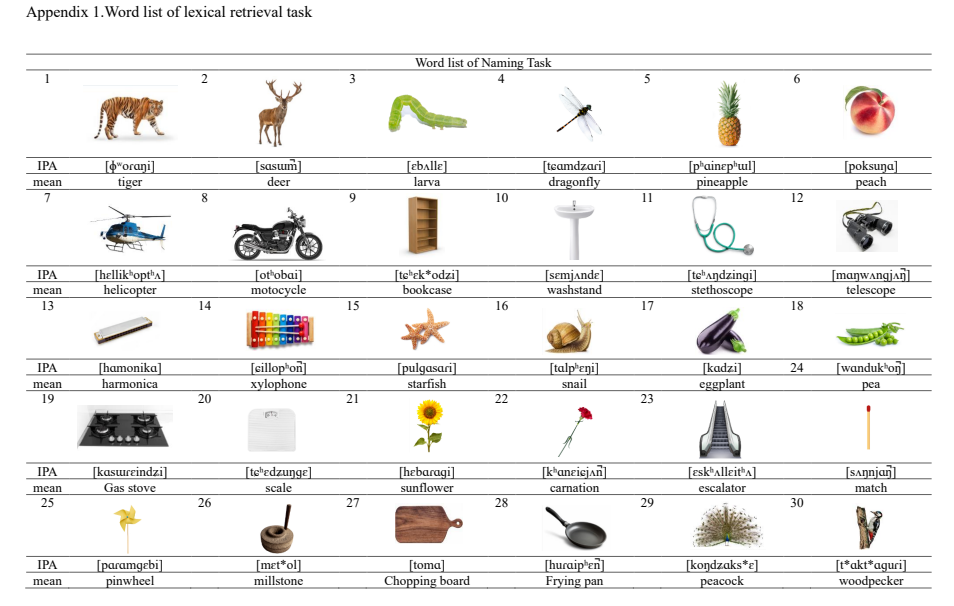


**Supplementary Appendix 2 Results of post-hoc power analysis**

| Statistical test | Variables | Effect size(d/f^2^) | Power(1-β) |
| --- | --- | --- | --- |
| Mann-Whitney U | NPH vs. HE (LRT score) | 2.46 | .99 |
| Mann-Whitney U | NPH vs. HE (FC) | 1.64 | .97 |
| Mann-Whitney U | NPH vs. HE (FD) | 1.68 | .97 |
| Mann-Whitney U | NPH vs. HE (SC) | 1.65 | .97 |
| Mann-Whitney U | NPH vs. HE (SD) | 1.86 | .99 |
| Mann-Whitney U | NPH vs. HE (SST) | 1.49 | .94 |
| Mann-Whitney U | NPH vs. HE (SAT) | 1.69 | .98 |
| Correlation | FC vs. LRT score | -.56 | .88 |
| Correlation | FD vs. LRT score | -.56 | .88 |
| Correlation | SC vs. LRT score | -.56 | .88 |
| Correlation | SD vs. LRT score | -.55 | .86 |
| Correlation | SST vs. LRT score | -.48 | .74 |
| Correlation | SAT vs. LRT score | -.46 | .68 |
| Two way mixed ANOVA  (within groups) | FC correct vs. FC Incorrect | 1.59 | 1.00 |
| Two way mixed ANOVA  (between groups) | NPH vs. HE  (FC correct /incorrect) | .56 | .88 |
| Two way mixed ANOVA (within groups) | FD correct vs. FD Incorrect | 1.39 | 1.00 |
| Two way mixed ANOVA (between groups) | NPH vs. HE  (FD correct /incorrect) | .58 | .91 |
| Two way mixed ANOVA (within groups) | SC correct vs. SC Incorrect | 1.61 | 1.00 |
| Two way mixed ANOVA (between groups) | NPH vs. HE  (SC correct /incorrect) | .57 | .90 |
| Two way mixed ANOVA (within groups) | SD correct vs. SD Incorrect | 1.98 | 1.00 |
| Two way mixed ANOVA (between groups) | NPH vs. HE  (SD correct /incorrect) | .75 | .98 |
| Two way mixed ANOVA (interaction effect) | Group * Response type | .49 | .99 |
| Two way mixed ANOVA (within groups) | SAT correct vs. SAT Incorrect | 1.38 | 1.00 |
| Two way mixed ANOVA (between groups) | NPH vs. HE  (SAT correct /incorrect) | .50 | .81 |
| Two way mixed ANOVA (within groups) | SST correct vs. SST Incorrect | 1.28 | 1.00 |
| Two way mixed ANOVA (between groups) | NPH vs. HE  (SST correct /incorrect) | .52 | .84 |

**Supplementary Appendix 3 Scatter plots of correlation analysis results** (A) Fixation count vs. Lexical retrieval task (B) Fixation duration vs. Lexical retrieval task (C) Saccade duration vs. Lexical retrieval task (D) Saccade count vs. Lexical retrieval task (E) Saccade scanpath total vs. Lexical retrieval task (F) Saccade amplitude total vs. Lexical retrieval task


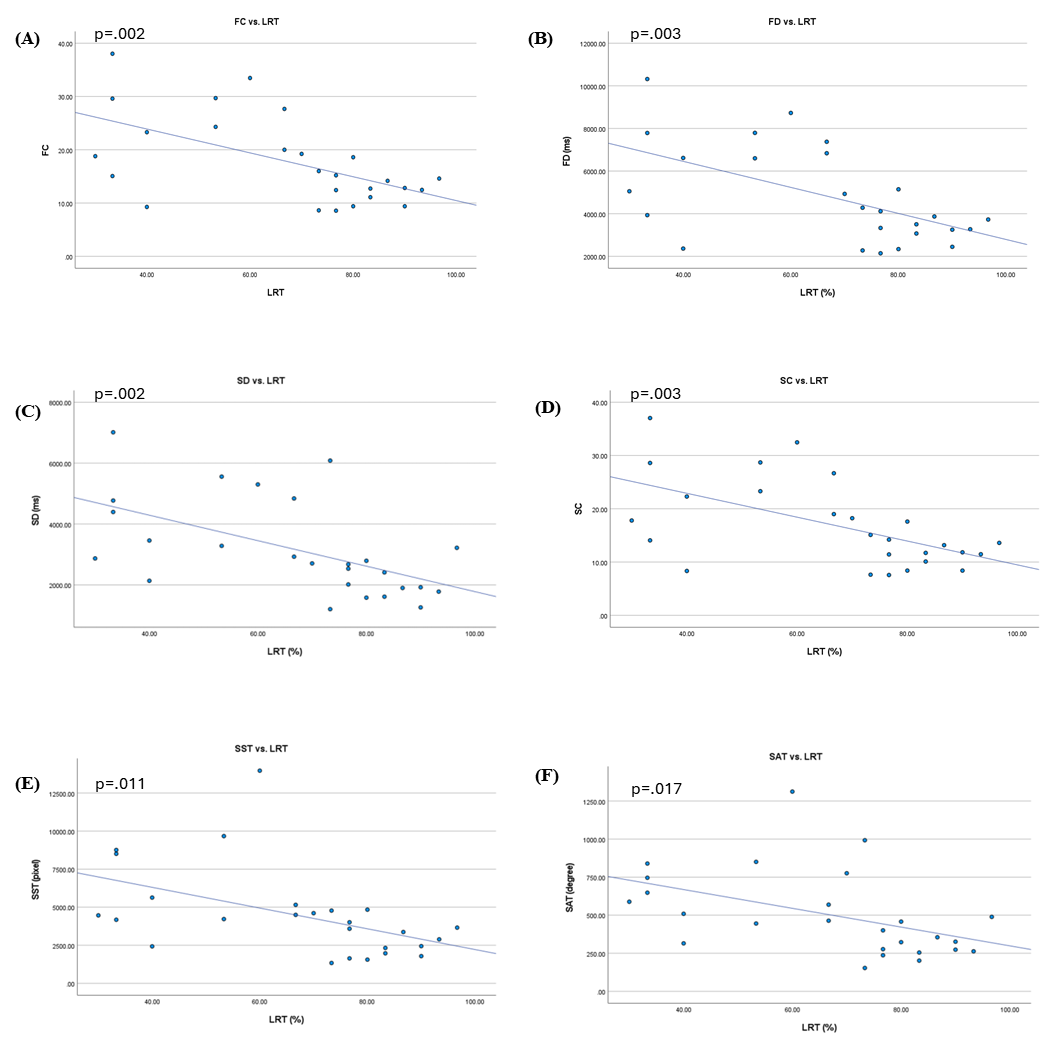


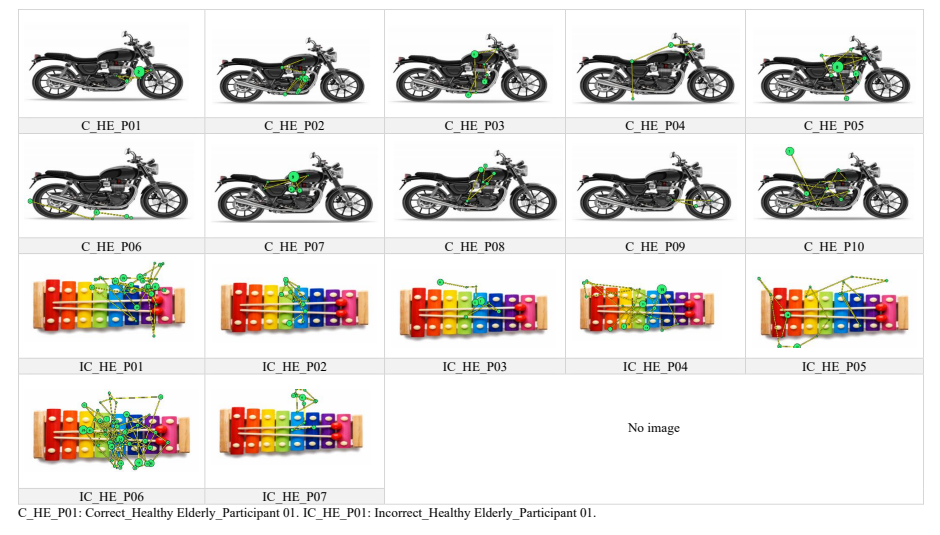
**Supplementary Appendix 4. Scanpaths for correct and incorrect items for both groups**


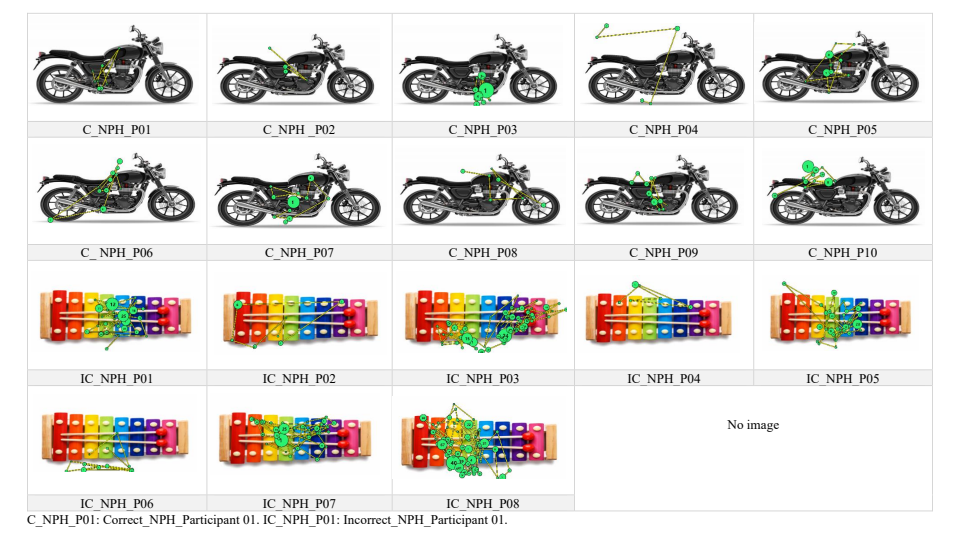


**Supplementary Appendix 5. A grid-based quantitative analysis between groups** (A) Correct response in ‘eggplant’ (B) Correct response in ‘motorcycle’ (C) Incorrect response in ‘xylophone’ (D) Incorrect response in ‘escalator’


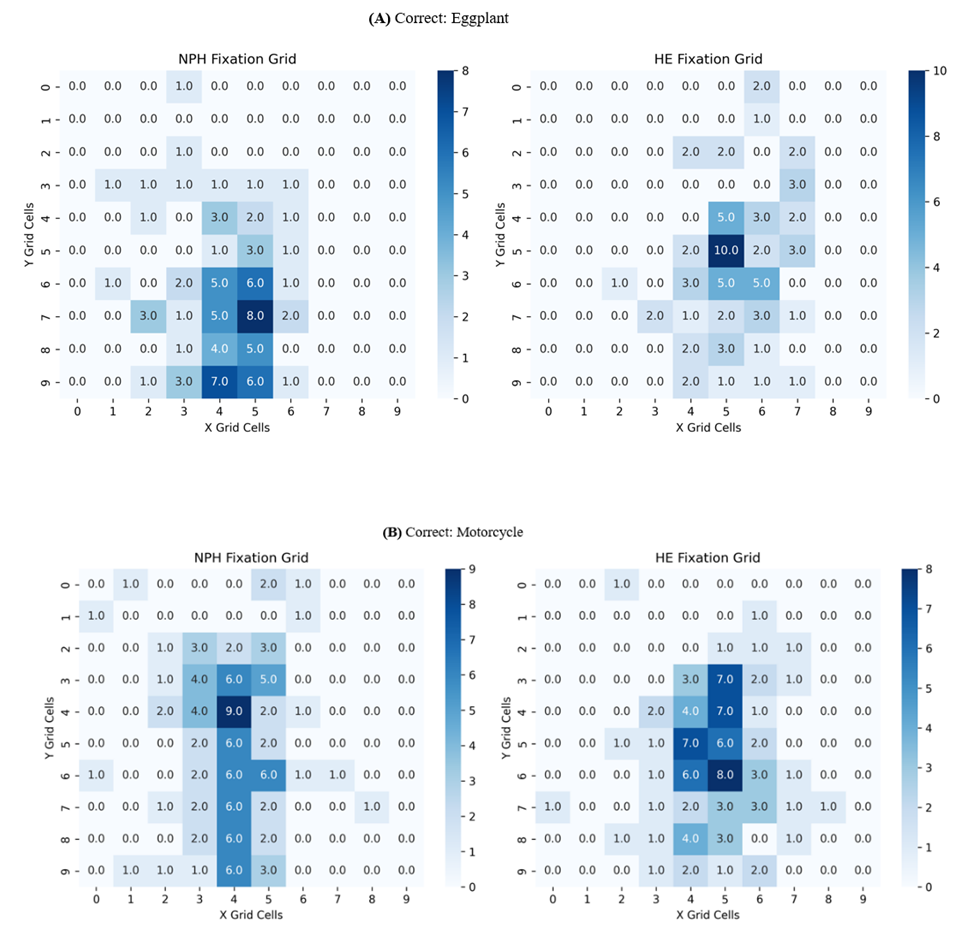


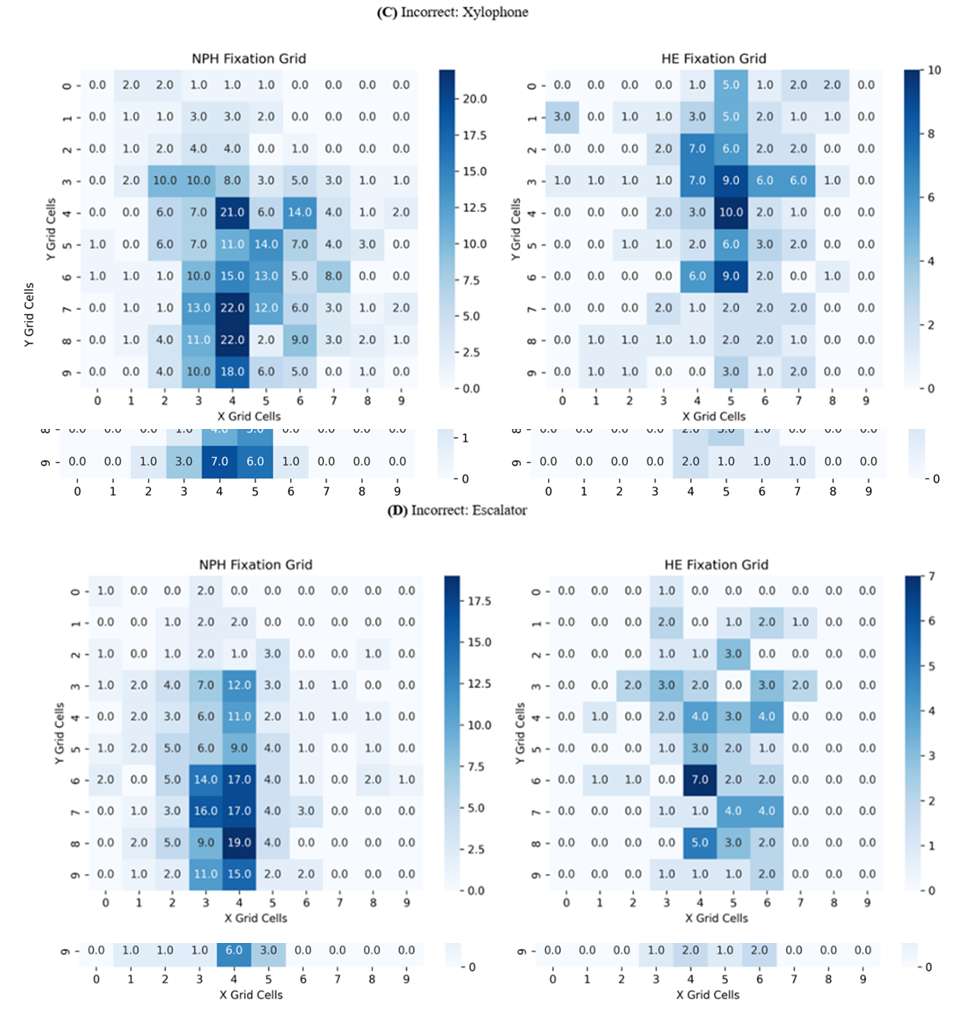

Supplement: Supplementary file 1 [file Data_Sheet_1.docx]
